# I Undervalue You but I Need You: The Dissociation of Attitude and Memory Toward In-Group Members

**DOI:** 10.1371/journal.pone.0032932

**Published:** 2012-03-07

**Authors:** Ke Zhao, Qi Wu, Xunbing Shen, Yuming Xuan, Xiaolan Fu

**Affiliations:** State Key Laboratory of Brain and Cognitive Science, Institute of Psychology, Chinese Academy of Sciences, Beijing, China; The University of Melbourne, Australia

## Abstract

In the present study, the in-group bias or in-group derogation among mainland Chinese was investigated through a rating task and a recognition test. In two experiments,participants from two universities with similar ranks rated novel faces or names and then had a recognition test. Half of the faces or names were labeled as participants' own university and the other half were labeled as their counterpart. Results showed that, for either faces or names, rating scores for out-group members were consistently higher than those for in-group members, whereas the recognition accuracy showed just the opposite. These results indicated that the attitude and memory for group-relevant information might be dissociated among Mainland Chinese.

## Introduction

The in-group bias, also called intergroup bias, usually refers to the phenomenon of in-group favoritism, which is a preference and affinity for in-group members over out-group members or anyone viewed as outside the in-group [1]. Studies showed that this phenomenon was robust that it could be demonstrated by many tasks, such as evaluation task, resource allocation task, attribution task, recognition test and many other ways [1]–[3]. For example, in the famous experiment conducted by Sherif and his colleagues [2], they found that campers exhibited consistent biases, namely they favored members of their own group over members of the competing group in the context of a boys' summer camp. The pervasive in-group favoritism was consistently reported on attitude and other cognitive dimensions [1]. It has positive meaning to people according to the social-categorization models [4].

The mainstream researches investigated the in-group favoritism phenomenon, and less work has been done to study a similar but completely opposite phenomenon: in-group derogation. However, this phenomenon was found in some special in-group members such as minorities and deviants. For example, the in-group derogation was found among the members of inferior groups like African Americans [5]. It was hypothesized that because they could not help but internalize society's biases against them and subscribe to negative views about their group in ways that justify the status quo and their group's occupation of an inferior social position, thereby affirming their belief in a just, predictable world [5]–[7]. In addition, the ‘black sheep effect’ shows that individuals derogate unlikable in-group members more negatively compared with their out-group counterparts [8]. Participants derogated deviant in-group members because in-group norms or values were undermined by them in a social context [9], [10].

In-group derogation phenomenon was mainly reported by cross-culture studies. For example, Kitayama, Palm, Masuda, Karasawa, and Carroll [11] found that Japanese viewed their own cities to be more vulnerable to earthquakes than those of Americans'. Snibbe and his colleagues [12] found that Americans showed in-group favoritism toward their own school's football teams, while Japanese did not. Recently, researchers reported that Mainland Chinese rated their intimate family members and friends less positively than Westerners [13], [14].

As shown above, the in-group derogation phenomenon seemed to be salient among East Asians. However, we found there were some problems within these studies. Firstly, the measuring instruments employed by previous studies [4], [13] seems to be reliable among Westerners, but there is no guarantee that they could work in Eastern cultures. In fact, no cross-culture studies have ever addressed this issue. The cross-culture validities of these instruments are practically unknown. Secondly, no researchers have ever investigated the in-group derogation by directly comparing the in-group and out-group members at the same time. Thus, the lower rating scores of the in-group members in Mainland Chinese might be caused by the appraisal criteria discrepancies between different cultures. As we know, the East Asians are more modest than Westerners [15], therefore, the lower scores of Chinese participants may be caused by their strict standard or their intrinsic modesty. Thirdly, researchers within this field have neither controlled the intensity of intergroup competition, nor the similarity and status difference between groups which will absolutely confound the results [16], [17].

To rule out the possibilities described above, we investigated the phenomenon of in-group bias or in-group derogation by adopting an improved methodology in which attitudes toward or memories of out-group members and in-group members were directly compared with each other while the status difference and similarity between them were well controlled [2], [3], [18]. We selected two universities (Beijing Forestry University, China Agriculture University) as two relatively equal status groups based on three reasons: Firstly, both of them belong to the National ‘211 Project’ universities. ‘211 Project’ is a project for building National Key Universities and colleges, and is initiated in 1995 by the Ministry of Education of the People's Republic of China, with the intent of raising the research standards of high-level universities and cultivating strategies for socio-economic development. Also, both of them have superior advantages over all other universities. The China Agricultural University is prominent in the domain of agriculture and Beijing Forestry University is famous for its major of forestry. Secondly, their criteria for admission are almost the same. Before entering the university, most Chinese high school students have to pass the National College Entrance Examination (NCEE), thus the average score for admitted students of each university is an important index of its quality. Table 1 shows the average scores and highest scores of the two universities for enrollment of students (in the Beijing area) in the latest four years. From these data, we can conclude that, as for natural sciences, the criterion for admission of China Agricultural University is a little higher than that of Beijing Forestry University; however, as for human sciences, the criterion for admission of Beijing Forestry University is a little higher than that of China Agricultural University. Thirdly, the geographical positions of the two universities are close. Both of the two universities locate in Haidian district, Beijing and the distance between two universities is about 1.6 kilometers (estimated from Google maps).

**Table 1:** Enrollment conditions for the two universities in the last four years.

Beijing Forestry University						China Agricultural University		
Natural Sciences			Human Sciences		Natural Sciences		Human Sciences	
Year	High	Average	High	Average	High	Average	High	Average
2010	650	572	623	561	650	584	605	564
2009	628	556	621	559	645	574	613	567
2008	632	558	611	555	653	578	579	553
2007	650	576	612	562	671	590	602	554

The average and highest scores for accepted students in Beijing in the last four years. ‘High’ indicates highest score and ‘Average’ indicates average score. The two universities are respectively named as Beijing Forestry University and China Agricultural University.

Previous studies consistently reported that the attitude toward and the cognitive superiority of in-group members were coherent [3], [18]. Based on this information, and considering both the faces and names are the best emblems of one's identity [19]–[22], we hypothesized that if the derogation of in-group members among Chinese really exists, then both the rating score and recognition accuracy for in-group members' faces and names would be lower than those of out-group members'.

## Methods

### Ethics statement

The experimental procedure was approved by the IRB of the Institute of Psychology, Chinese Academy of Sciences. All participants provided written, informed consent before taking part in our experiments.

### Experiment 1

#### Participants and Design

Thirty undergraduates from China Agricultural University (18 females) and thirty undergraduates from Beijing Forestry University (18 females) participated in this experiment as paid volunteers. A 2 (subject variable: subjects of China Agricultural University (SCA), subjects of Beijing Forestry University (SBF)) ×2 (category label: In group, out group) mixed-model experimental design was used, with repeated measures on the second factor.

#### Materials and Procedure

**Figure 1:**
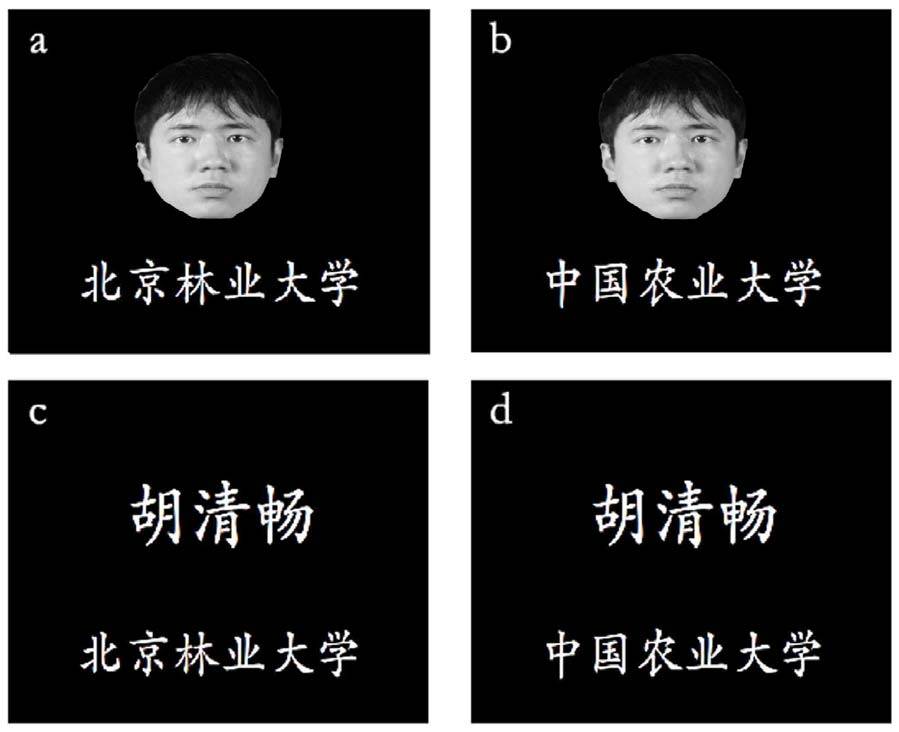
Sample of stimuli in experiment 1 and 2. Examples of stimuli in the experiment 1 (a, b) and experiment 2 (c, d) or three-character Chinese name (c, d) was presented at the center of screen. Beijing Forestry University name (a, c) or China Agricultural University name (b, d) was inscribed at the bottom of screen.

Eighty gray-scale facial images of Chinese college-age males and females with neutral expressions were chosen from three Asian facial expression databases [23]–[25] as the stimuli set which were completely novel to all participants. The stimuli were randomly divided into two sets and thirty-two college students who did not participate in the formal experiments rated the degree of beauty on a ten point scale (1 =  ‘not beautiful at all’ to 10 =  ‘extremely beautiful’) for each set. Paired *t*-test showed that for the degree of beauty there was no difference between first set (*M* = 5.57, *SD* = 0.45) and the second set (*M* = 5.58, *SD* = 0.47), *t*(31) = 0.491, *P*>0.05. As for the thirty participants from Beijing Forestry University, fifteen of them were exposed to a stimuli sequence in which the first facial image set was labeled with their Alma mater name and the second image set was labeled with other university name (China Agricultural University). The other fifteen were exposed to a stimuli sequence in which the second facial image set was labeled with their Alma mater name whereas the first image set was labeled with the other university name. The same procedure was employed for the thirty participants from China Agricultural University, thus each image set had the equal probability to be labeled as in-group or out-group members. Adobe Photoshop was used to crop the face and resize the images to 210×273 pixels. Each face was presented at the center of the computer screen on a black background with the resolution of 640×480 pixels. The university name was placed at the bottom of the background in order to label the face (as shown in Fig. 1).

After providing informed consent, subjects were seated in front of the computers and were instructed that they would complete an appraisal task and a recognition test. All instructions and stimuli were presented via computers. Subjects were told that the faces were selected from two university rosters and that there might be some difference in the degree of beauty between two universities. They were instructed that, during appraisal phase, they would see 80 faces labeled with university names, and their task was to remember which university did the faces come from and then to select a number from 1 to 10 for each face according to its degree of beauty by clicking the mouse. Each face was presented for 3500 ms. After the subject's response, a black screen appeared for a randomized duration from 2000 to 2500 ms. Sequence of the faces were randomized for each subject. Subjects were then engaged in the recognition phase after a five minutes' rest. They were instructed that they would see a series of faces labeled with university names, some of which they had seen during the appraisal phase (old faces) and some of which they had not seen (new faces). Subjects were instructed that as each face appeared on the screen, they should press a button to indicate whether or not they had seen it during appraisal phase. Each face remained on the screen until the decision was made. A black screen was presented for 2000 to 2500 ms between the two trials during this phase. Eighty faces were presented at the recognition phase. Forty faces were old faces (20 faces labeled with China Agricultural University names and 20 faces labeled with Beijing Forestry University names) and forty were new faces (20 faces labeled with China Agricultural University names and 20 faces labeled with Beijing Forestry University names). During the recognition phase, the faces appeared in a random order.

### Experiment 2

#### Subjects and Design

Thirty undergraduates from Beijing Forestry University (16 females) and thirty undergraduates from China Agricultural University (16 females) participated in this experiment as paid volunteers. A 2 (subject variable: subjects of China Agricultural University (SCA), subjects of Beijing Forestry University (SBF)) ×2 (category label: In-group, out-group) mixed-model experimental design was used, with repeated measures on the second factor.

#### Materials and Procedure

Eighty three-character Chinese names (containing both family and first name) of Chinese were used as the stimuli. Just like in Experiment 1, we randomly divided the names into two different sets and thirty-two college students who did not participate in the formal experiments rated the degree of catchiness on a ten point scale (1 =  ‘not catchy at all’ to 10 =  ‘extremely catchy’) for each set. Paired *t*-test showed that for the degree of beauty there was no difference between first set (*M* = 5.74, *SD* = 0.59) and the second set (*M* = 5.73, *SD* = 0.55), *t*(31) = 0.29, *P*>0.05. The stimuli were generated in the same way as in Experiment 1. The Chinese names were displayed with a resolution of approximately 320×115 pixels, and each name was then placed on the black background with a resolution of 640×480 pixels. The university name was presented in white at the bottom of the background (as shown in Fig. 1).

The procedure was identical to that of Experiment 1, except that subjects were asked to rate the degree of catchiness for each name in the appraisal phase.

## Results

### Experiment 1

**Figure 2:**
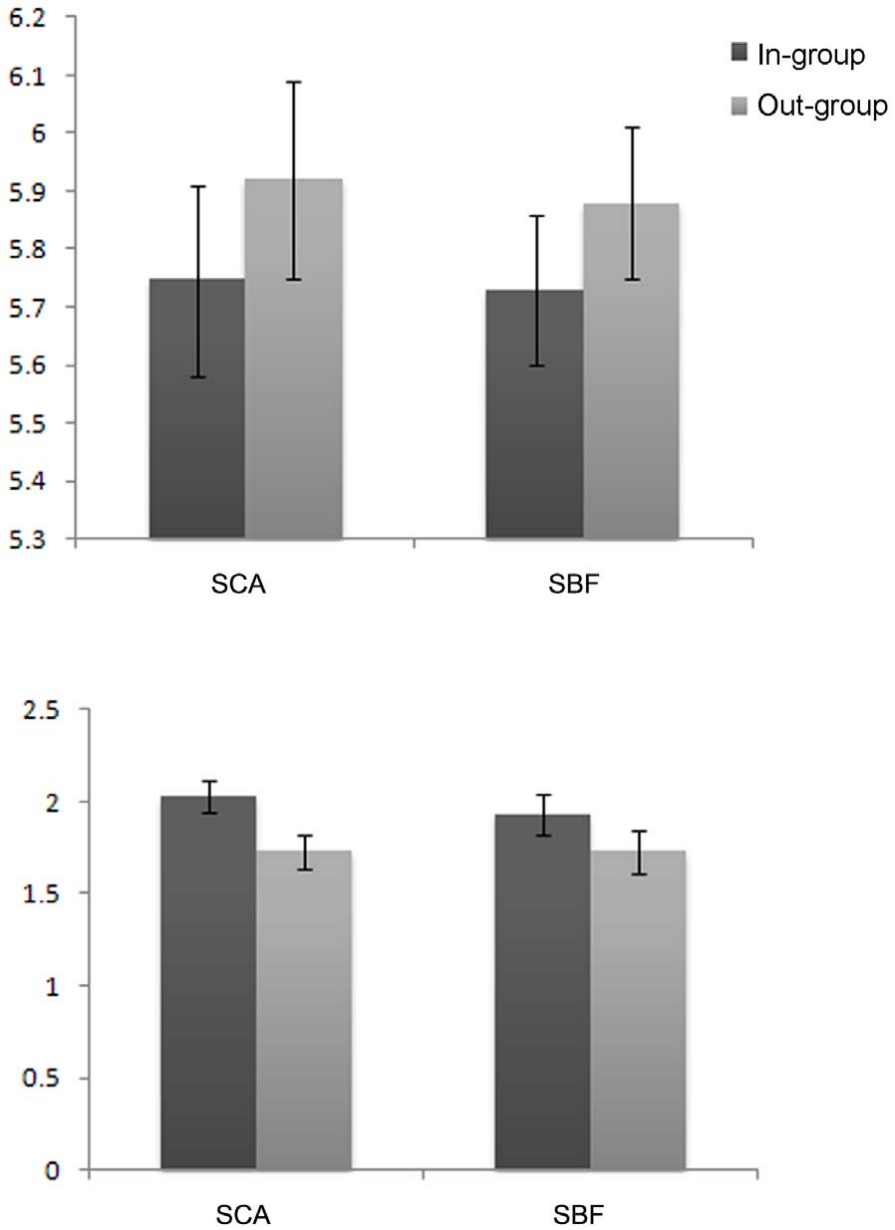
Results of experiment 1. Rating scores (top) and recognition accuracy (bottom) for faces labeled in-group university name and out-group university name for subjects from China Agricultural University (SCA) and subjects from Beijing Forestry University (SBF) in the experiment 1.

It is interesting to see to what extent the presence of the group labels will influence the face appraisal process and face recognition. To test whether the mere exposure of social-category labels influenced beauty appraisal, the rating scores were subjected to a 2 (subject variable: SCA, SBF) ×2 (category label: in-group, out-group) mixed-model analysis of variance (ANOVA), with repeated measures on the second factor. The main effect of category label was significant, *F*(1, 59) = 8.67, *P*<0.05, *η_p_* ^2^ = 0.13 (see Fig. 2). The faces labeled with subjects' Alma mater names were appraised lower (*M* = 5.42, *SD* = 0.94) than faces labeled with other university names (*M* = 5.53, *SD* = 0.92). However, results showed that the main effect of subject variable [*F*(1, 59) = 0.24, *P*>0.05, *η_p_* ^2^ = 0.01] and interaction between category label and subject variable [*F*(1, 59) = 1.62, *P*>0.05, *η_p_* ^2^ = 0.02] were not significant. As predicted, the rating scores for out-group faces were higher than those for in-group faces. This result indicated that when familiarity was controlled, subjects inclined to derogate the degree of beauty for faces of in-group members.

To test whether the mere presence of social-category labels would influence the face recognition, we calculated the recognition accuracy rate for each face. The sensitivity (d′) was chosen as the performance index. We subjected sensitivity scores to a 2 (subject variable: SCA, SBF) ×2 (category label: in group, out group) mixed-model analysis of variance (ANOVA), with repeated measures on the second factor. The main effect of category label was significant, *F*(1, 59) = 8.45, *P*<0.05, *η_p_* ^2^ = 0.12. The recognition performance for the faces of subjects' in-group members (*M* = 1.50, *SD* = 0.58) was higher than the faces from out-group (*M* = 1.33, *SD* = 0.54). However, the main effect of subject variable [*F*(1, 59) = 0.04, *P*>0.05, *η_p_* ^2^ = 0.01] and interaction between category label and subject variable [*F*(1, 59) = 1.77, *P*>0.05, *η_p_* ^2^ = 0.03] were not significant. Out of our expectation, the better performance was obtained for the faces labeled with Alma mater name than those labeled with other university name.

More importantly, we found no significant correlation between rating score and recognition performance for neither in-group faces (*r* = 0.19, *p* = 0.13) nor out-group faces (*r* = 0.10, *p* = 0.44), indicating that two processes were independent.

### Experiment 2

**Figure 3:**
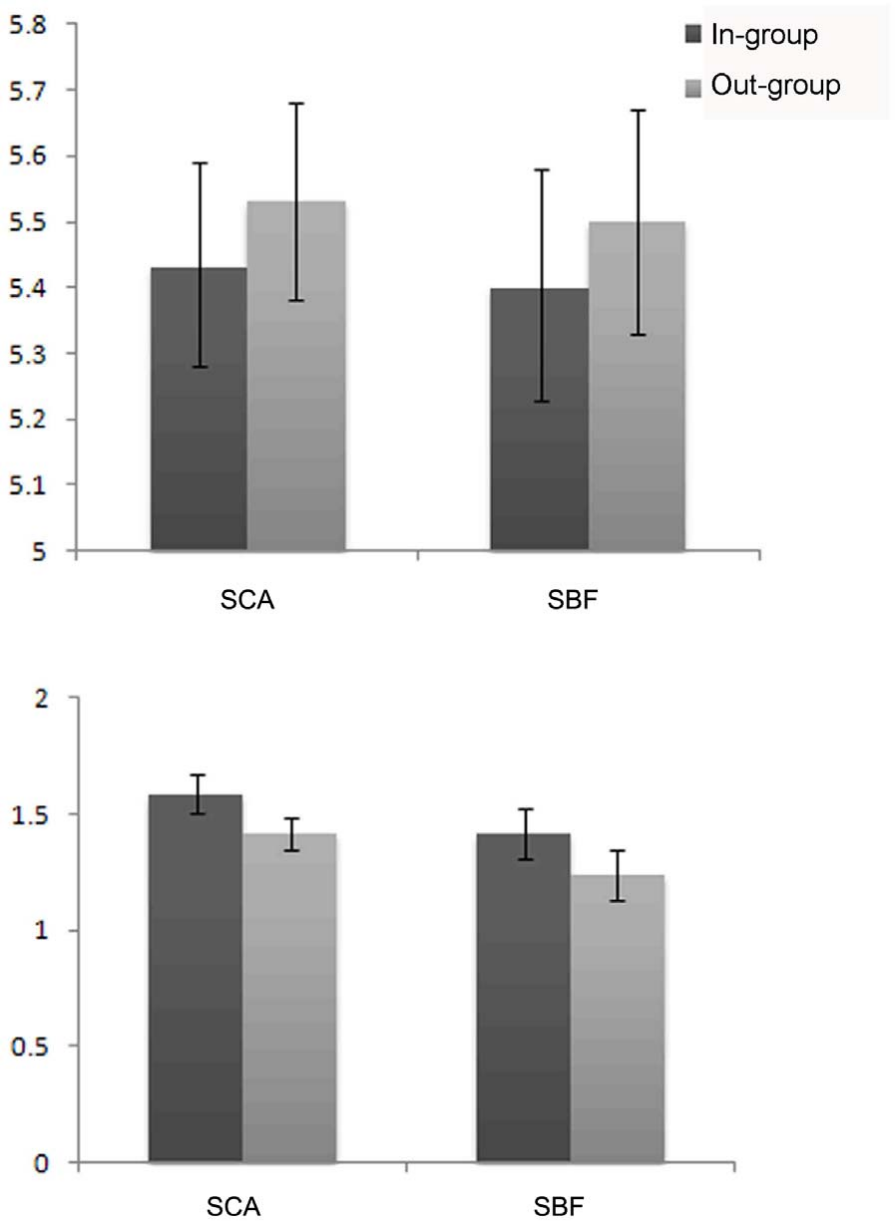
Results of experiment 2. Rating scores (top) and recognition accuracy (bottom) for names labeled in-group university name and out-group university name for subjects from China Agricultural University (SCA) and subjects from Beijing Forestry University (SBF) in the experiment 2.

We conducted similar ANOVA to the results of experiment 2. The main effect of category label was significant, *F*(1, 59) = 13.20, *P*<0.05, *η_p_* ^2^ = 0.22 (see Fig. 3). The names labeled as in-group members were appraised lower (*M* = 5.74, *SD* = 0.82) than those labeled as out-group members (*M* = 5.90, *SD* = 0.84). The main effect of subject variable [*F*(1, 59) = 0.01, *P*>0.05, *η_p_* ^2^<0.01] and interaction between category label and subject variable [*F*(1, 59) = 0.02, *P*>0.05, *η_p_* ^2^<0.01] were not significant. Consistent with Experiment 1, the rating scores were higher for out-group names than for in-group names. Subjects inclined to underrate the degree of catchiness for in-group members.

For the recognition performance, the main effect of category label was significant, *F*(1, 59) = 17.09, *P*<0.05, *η_p_* ^2^ = 0.23. The recognition performance for the names of subject's in-group members (*M* = 1.98, *SD* = 0.60) was higher than those of out-group members (*M* = 1.73, *SD* = 0.53). However, the main effect of subject variable [*F*(1, 59) = 0.12, *P*>0.05, *η_p_* ^2^<0.01] and interaction between category label and subject variable [*F*(1, 59) = 0.65, *P*>0.05, *η_p_* ^2^ = 0.01] were not significant. Consistent with Experiment 1, the recognition performance of in-group member's names was better than that of out-groups'. The detachment between appraisal and recognition for the in-group relevant information were replicated in Experiment 2.

Again, there was no significant correlation between rating score and recognition performance for neither in-group names (*r* = 0.07, *p* = 0.62) nor out-group names (*r* = 0.16, *p* = 0.22). These results indicated that two processes should be dissociated.

## Discussion

By directly comparing rating scores for in-group and out-group members, our two experiments consistently showed that, when the intensity of competition and similarity between groups were controlled, sustaining lower rating scores of both faces and names were given to in-group members rather than out-group members. The results demonstrated that the phenomenon of in-group derogation might be ubiquitous and the attitude toward in-group members was more negative than toward out-group members. Surprisingly, the recognition accuracies of the names and faces labeled as in-group members were higher than those labeled as out-group members. This result was consistent with previous studies which supported that in-group relevant information rather than out-group relevant information would escalate the memory performance [18], [26]. In summary, our results indicated that the attitude toward and the memory for in-group members were not always coherent.

How could this happen? According to the social category model, the in-group relevant information will be granted with more holistic processing than out-group relevant information [27]–[29], which facilitates the encoding, and results in better memory performance. The other theoretical model of self-categorization considers that the significance of in-group members to us will result in deep and individualized process of in-group information rather than out-group information [30], [31]. Thus, the recognition of in-group members will be better than that of out-group members. This theory was also supported by the functional magnetic resonance imaging (fMRI) studies, for example, Golby, Gabrieli, Chiao, and Eberhardt [32] reported a direct correlation between fusiform activity and recognition memory, such that participants with the largest difference in recognition memory of own-race faces compared with other-race faces also had the largest difference in fusiform activity to own-race faces compared with other-race faces; Van Bavel, Packer, and Cunningham [33] further found that the fusiform activity was closely related to the recognition performance of in-group information. However, the attitude favoritism and cognitive priority to in-group relevant information were always found to be coherent [24], [25], [34]–[36]. Existing fMRI studies indicated that the attitude and motivation related to the memory of in-group members were disassociated. Van Bavel, Packer, and Cunningham [3] found that one's attitude was mediated by the orbitofrontal cortex, when an individual prefers in-group members the stronger activation was elicited. However, they [33] found that fusiform gyri were responsible for the top-down motivation of in-group members, the stronger the motivation, the better the memory performance. The result of dissociation between rating and recognition for in-group members supported that there were separated mechanisms to process the attitude and memory of group-relevant information. Besides, our clear-cut results indicated that though we derogated the in-group members, the recognition superiority for in-group members still remained.

Why we undervalue the in-group members? According to injustice theory, minorities subscribe to negative views about their group in ways that justify the status quo and their group's occupation of an inferior social position. Consequently, in-group derogation should be limited to dimensions that are status-relevant (e.g., intelligence), not status-irrelevant (e.g., enthusiasm) [5], [7], [37]. However, in our studies, though the content of appraisal task was status-irrelevant and the two universities we selected had the equal rank, the phenomenon of in-group derogation still emerged. Similar evidence was also obtained by another research which reported whether Chinese were as a majority (high status) in Malaysia or minority (low status) in Singapore, no in-group favoritism emerged in attribution tasks [38]. Meanwhile, in Ma-Kellams, Spencer-Rodgers, and Peng [14] researches (studies 2 & 3), they used Implicit Association Test (IAT) to investigate the in-group derogation among Chinese. Their results also indicated that the in-group derogation among Chinese is also irrelevant to status. In the western cultures, there exists ‘black sheep effect’ which means that individuals derogate unobservant in-group members compared with the same type of members in out-group [8]. Because our study controlled the variable of group norm, the in-group derogation cannot be attributed to in-group members whom we seemed as deviants.

As we know, culture influences our cognition style, especially in the domain of social cognition [39]. Based on the discussions mentioned above, we speculate that the in-group derogation phenomenon found among Eastern Asians might be a result of culture difference, though what specific difference is still unclear. It should be noted that the dialectic theory also reckoned that the derogation of in-group members among East Asians was a result of the culture difference. As Ma-Kellams, Spencer-Rodgers, and Peng [14] have explained, the Chinese hold a dialectical belief while Westerners hold a linear belief. People in China incline to appraise both the bad and good for the same object, but westerners mainly see the good. Thus in their studies, Chinese appraised their family members more negatively than westerner in the same dimensions. This dialectic theory can explain why the criteria of appraisal for Chinese are stricter than the Western folk, but cannot explain the lower rating score for the in-group members compared to the out-group members in the present study, because in our experiments the participants were from the same-race (all were Chinese). Based on the dialectic theory, they would employ the same dialectical belief to look upon the in-group and out-group members, then this same appraisal criterion would have resulted in equal rating scores for in-group and out-group members. Another possible explanation is that modesty, as a possible trait of East Asians, might lead Chinese to undervalue in-group members [40]. Because there have not been enough studies to systematically explore the relationship between in-group derogation and modesty, the conclusion is still early to draw.
